# When Standard Therapy Fails in Breast Cancer: Current and Future Options for HER2-Positive Disease

**DOI:** 10.4172/2167-0870.1000129

**Published:** 2013-03-06

**Authors:** Erin Olson, D’Anna Mullins

**Affiliations:** Clinical Fellow, Divisions of Medical Oncology and Hematology, The Wexner Medical Center at the Ohio State University, USA

**Keywords:** Trastuzumab, HER2 therapies, Phase III trials

## Abstract

The area of HER2-positive breast cancer is a rapidly changing field. The use of the humanized monoclonal antibody, trastuzumab, significantly improved the prognosis for patients with HER2-positive breast cancer, however, increasing knowledge regarding mechanisms of resistance to trastuzumab have come to light, prompting research into additional methods to target the HER2 protein. The purpose of this article is to discuss evidence for why continued blockade of the HER2 pathway continues to be important despite progression on trastuzumab, as well as to review additional HER2-targeted therapies and progression in the central nervous system. With the availability of new drugs comes the need to determine the appropriate therapeutic combinations and optimal order in which to deliver these therapies. This review summarizes the practice-changing phase III trials and some supporting phase II data regarding the various targeted HER2 therapies available for patients with advanced HER2-positive breast cancer, proposes order for anti-HER2 therapy in the advanced HER2-positive breast cancer patient, and includes information on future strategies. While other reviews on HER2-targeted therapy are available, this review specifically aims at addressing treatment options after trastuzumab failure in the patient with advanced HER2-positive breast cancer.

## Introduction

The Human Epidermal Growth Factor Receptor 2 (HER2) oncogene is responsible for encoding a transmembrane tyrosine kinase which is over expressed in 20–25% of breast cancers and is associated with a worse prognosis [1,2]. The use of trastuzumab, a humanized monoclonal antibody to the HER2 extracellular domain, has been shown to improve survival in both early and metastatic HER2-positive patients [3,4], and is now the standard of care in this patient population. Despite these advances, approximately 15% of patients treated with adjuvant trastuzumab will relapse [5,6], and progressive disease often occurs after first-line trastuzumab therapy in the metastatic setting within 12 months [5,6]. These limitations to our current repertoire of anti-HER2 therapy have led to the rapid development of next-generation HER2-targeted agents aimed at inhibiting mechanisms of acquired resistance to trastuzumab.

**Figure 1:**
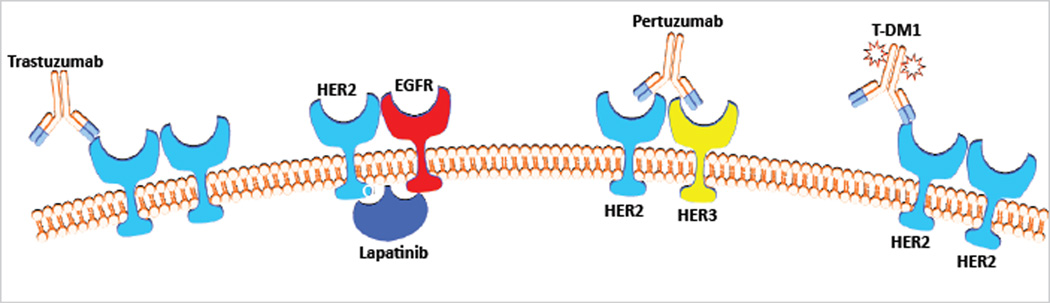
HER2-targeted drugs. Trastuzumab, Trastuzumab, lapatinib and pertuzumab are currentlyFDA-approved. HER: human epidermal growth factor receptor; EGFR: epidermal growth factor receptor; T-DM1: trastuzumab emtansine.

Recent evidence suggests that activation of the HER2 signaling pathway continues to be the primary driver of tumor growth and survival, even after prolonged exposure to trastuzumab. Multiple resistance mechanisms have been proposed, and include up-regulation of ligand-induced receptor activation, increased signaling from other HER family receptors, and activation of the phosphatidylinositol triphosphate kinase (PI3K) pathway with loss of phosphatase and tensin homolog (PTEN) [5–8]. Given this information, there has been a surge of research in the area of HER2-positive breast cancer, studying new mechanisms of anti-tumor drug delivery (i.e. T-DM1), as well as novel therapeutic combinations (i.e. trastuzumab, pertuzumab and docetaxel). The breast cancer practitioner can partake in several different approaches when treating progressive HER2-positive disease. Examples of these clinical tactics to overcoming trastuzumab resistance include continuation of trastuzumab with a alternative chemotherapy backbone, switching to a different targeted therapy such as lapatinib or pertuzumab, as well as combining two HER2-targeted agents with other agents. The oncology community has made vast strides in the treatment of HER2-amplified breast cancer; however, the question of the optimal order and duration of therapies remains in flux. This review will address therapeutic options for HER2-positive breast cancer patients who progress after trastuzumab, attempting to outline the most favorable algorithm for clinicians utilizing FDA-approved regimens as well as novel therapeutics that are currently under study (Figure 1).

## Trastuzumab after Progression

Trastuzumab is a humanized monoclonal antibody which binds to the extracellular domain of the HER2 molecule [9]. Proposed mechanisms of action include antibody-dependent cellular cytotoxicity, inhibition of intracellular signal transduction, inhibition of tumor angiogenesis, and inhibition of repair of DNA damage induced by concurrent chemotherapy [8]. Notably, removal of trastuzumab routinely results in tumor progression [10]. Prior to trastuzumab, the general practice in oncology was to switch to a different regimen upon documented growth of metastatic tumor lesion. Over the last decade, treating oncologists have routinely continued trastuzumab beyond progression – well before there was any prospective data to support this practice – leading to an unprecedented treatment paradigm in the solid-tumor arena. One of several retrospective trials investigating the benefit of continuation of trastuzumab was a multicenter, international study which identified 105 patients with metastatic HER2-positive breast cancer who had received trastuzumab after documented progression on a trastuzumab-containing regimen [11]. Their analysis revealed a 39% overall response rate with trastuzumab monotherapy or combined with chemotherapy in the second-line setting [11]. Additionally, patients who did not respond to a first-line regimen could engender a durable response to a second trastuzumab-containing regimen [11], and these findings were replicated in other retrospective studies suggesting that prolonged HER2-blockade may be efficacious in this patient population [12].

The German Breast Group 26/Breast International Group 03–05 trial was the first and only randomized, prospective trial to evaluate the efficacy of use of trastuzumab beyond progression on a previous trastuzumab-containing regimen [13]. Patients with locally advanced or metastatic HER2-positive breast cancer were randomized to either continue trastuzumab with the addition of capecitabine or to receive capecitabine alone. As stated prior, continuation of trastuzumab beyond progression was already common practice in both the United States and Europe, resulting in difficulty to meet the target accrual goal of 482 patients in this trial. The investigators were forced to close the study after enrolling only 156 patients. Despite these barriers, the authors reported a significantly improved median time to progression (5.2 months in the capecitabine monotherapy arm compared to 8.2 months in the combination arm), as well as a significantly improved overall response rate of 27% with capecitabine monotherapy compared to 48.1% in the combination arm [13]. Overall survival rates were not significantly different, likely as a result of poor accrual and nearly half of the participant’s crossing over to the trastuzumab arm in this intent-to-treat analysis. Importantly, continued use of trastuzumab was not accompanied by a significant increase in toxicity.

An additional *post-hoc* analysis of this study was performed comparing continued HER2-targeted therapy as second or third line therapy in the form of trastuzumab or lapatinib versus chemotherapy alone [14]. In this analysis, there was a statistically significant post-progression survival improvement from 13.3 months to 18.8 months. Although the numbers are small, these data support the continuation of trastuzumab despite progression on a trastuzumab-containing regimen, and given the current clinical practice, a trial like this will likely never be repeated.

## Lapatinib

The first HER2-targeted therapy to receive FDA approval after trastuzumab was lapatinib. Lapatinib is a small molecule, oral tyrosine kinase inhibitor of both HER2 and Epidermal Growth Factor Receptor (EGFR), and was initially shown in pre-clinical models to be effective in tumors resistant to trastuzumab [15,16]. A phase III clinical trial compared lapatinib plus capecitabine to single-agent capecitabine in women with locally-advanced or metastatic HER2-positive breast cancer [17]. The interim analysis prompted termination of enrollment on the trial, as there was a 51% reduction in disease recurrence risk and median time to progression was doubled (8.4 months with combination therapy versus 4.4 months with single-agent therapy) at that time [17]. Common adverse effects which were statistically more common in the combination arm were diarrhea, dyspepsia and rash; however, treatment discontinuations were even between the groups (13% in the combination arm versus 12% in the single-agent arm) [17]. Final survival analysis of this study data did not meet statistical significance, but there was a trend toward improved survival with the addition of lapatinib to capecitabine [18]. Because the trial was stopped early, the investigators were provided only a 68% statistical power to detect 30% overall survival difference versus a 90% power, had the trial completely accrued [18]. Given this information, statistical attempts at adjusting for crossover were made, resulting in a 20% lower risk of death in the combination arm; despite these hurdles, the addition of lapatinib to capecitabine in HER2-positive breast cancer patients continued to be supported [18].

Lapatinib is also FDA-approved in combination with letrozole as first-line treatment for post-menopausal patients with metastatic HER2-positive, estrogen receptor positive breast cancer based on a phase III randomized placebo-control trial [19]. Over 1200 patients were enrolled, and patients who received lapatinib in addition to letrozole had a 29% decreased risk of disease progression than the patients who received letrozole alone [19]. The combination was also well-tolerated, reporting an increase in grade 1 and 2 diarrhea and rash in the group receiving lapatinib, with only rare grade 3 events [19].

Additional studies performed with lapatinib in combination with trastuzumab in the metastatic setting after progression on trastuzumab have been performed. Blackwell et al. performed a phase III randomized international trial comparing lapatinib alone to the combination of lapatinib and trastuzumab in 296 patients [20]. Patients were allowed to cross-over from the lapatinib to the combination arm if they showed progression after 4 weeks; the number of patients who crossed over was 73 (49%). Progression free survival was increased from 8.1 weeks to 12 weeks with the combination of lapatinib and trastuzumab (*p*=0.008) [20]. Additionally, twice the number of patients who were progression free at 6 months was seen in the combination arm compared with the lapatinib only arm (28% vs. 13%) [19]. There was also a trend toward combination therapy providing an improved overall survival, as median overall survival during the study at the time of this analysis was 51.6 weeks in the combination arm and only 39 weeks in the lapatinib only arm. After adjusting for performance status, number of metastatic sites and presence or absence of liver involvement, there was a 27% reduction in risk of progression with the combination of lapatinib and trastuzumab compared to lapatinib alone [20]. With regards to adverse events, grade 1 and 2 diarrhea was present more significantly in the combination arm (60%) compared to the lapatinib only arm (48%), however, grade 3 or 4 diarrhea was not different between the two groups [20]. Rash was seen more in the lapatinib only arm. A 20% decrease from baseline in left ventricular ejection fraction was seen in 8 patients in the combination arm compared to 3 patients in the lapatinib only arm. Most of these were asymptomatic and transient decreases. Final planned overall survival analysis revealed a continued benefit of combination therapy over lapatinib alone, with an increase in absolute overall survival rates of 10% at 6 months and 15% at 12 months, and a 4.5 month improved median survival [21]. It is important to note that this 26% reduction in risk of death was statistically significant, and was present despite cross-over of almost 50% of patients from the lapatinib only arm to the combination arm [21].

Lapatinib is an active agent in patients with evidence of progression on trastuzumab, and is a safe and tolerable second-line anti-HER2 drug. It is important to note that it is unknown what – if any – activity lapatinib will have after progression on other next-generation HER2-targeted therapies such as pertuzumab and T-DM1, and further clinical trials to determine the correct timing of lapatinib therapy are warranted.

## Pertuzumab

The next agent approved for metastatic HER2-positive breast cancer was pertuzumab. Pertuzumab is a humanized monoclonal antibody which binds to the dimerization domain of HER2, thereby inhibiting dimerization with the other members of the HER family of receptors. In the international, randomized, double-blind, phase III CLEOPATRA trial, the addition of pertuzumab to trastuzumab plus docetaxel was studied in patients with newly metastatic HER2-positive breast cancer, 40–47% of who received previous trastuzumab [22]. The addition of pertuzumab improved progression-free survival from 12.4 months to 18.5 months (HR 0.62, 95% confidence interval, 0.51 to 0.75, p<0.001) [22]. This effect was also seen in patients who had prior exposure to trastuzumab in the adjuvant or neoadjuvant setting [22]. The combination of two HER2-targeted therapies did not increase the incidence of left ventricular systolic dysfunction significantly; however, there were higher rates of grade 3 febrile neutropenia and diarrhea in the patients receiving pertuzumab [22]. Updated survival data from this study was presented at the 2012 San Antonio Breast Cancer Symposium at 69% of overall survival events reached [23]. The results continued to show a statistically significant decrease in risk of death with the use of pertuzumab, trastuzumab and docetaxel over trastuzumab and docetaxel alone of 34% [23]. Median overall survival had not yet been reached in the pertuzumab, trastuzumab, docetaxel arm as of the time of this second interim overall survival analysis [23]. This study has been practice-changing, leading to the combination of trastuzumab, pertuzumab and docetaxel as the first-line choice for newly metastatic HER2-positive breast cancer patients.

Pertuzumab was also studied in combination with trastuzumab, without the addition of chemotherapy. A phase II study evaluated the combination of pertuzumab and trastuzumab in HER2-positive breast cancer patients after progression on a trastuzumab-based therapy in the metastatic setting. An overall response rate of 24.2% and a clinical benefit rate of 50% were observed [24]. Median progression-free survival was 5.5 months, boasting a durable clinical benefit [24]. Again, no patients had to withdraw due to cardiac-related adverse events, and a decrease in left ventricular ejection fraction was only seen in 3 of 66 patients. Gastrointestinal (diarrhea, nausea) and skin toxicities were the most frequent adverse events, and most were grade 1 or 2 [24]. A third cohort of 29 patients was recruited to this study to assess efficacy of pertuzumab monotherapy, as well as subsequent reintroduction of trastuzumab after progression on pertuzumab [25]. All 29 patients progressed on pertuzumab monotherapy, with a clinical benefit rate of 10%. Seventeen patients went on to receive the addition of trastuzumab to pertuzumab; in this population, objective response rate was 17.6% with a clinical benefit rate of 41.2% [25]. Although this study was a small phase II study, the results add to existing data to support the continuation of HER2-targeted therapy past progression on either agent, and the trial also suggests the clinical benefit of combining agents with complimentary mechanisms of HER2 blockade.

## Trastuzumab Emtansine (T-DM1)

T-DM1 is the most recent anti-HER2 agent found to have considerable activity in patients with trastuzumab-resistant disease. T-DM1 is an antibody-drug conjugate which links trastuzumab to DM1 via a thioether linker called *N*-maleimidomethyl Cyclohexane-1-Carboxylate (MCC). This linker is thought to help isolate exposure of DM1 to the target cells and spare normal tissue from the toxicity associated with cytotoxic chemotherapy. DM1 is a derivative of maytansine 1, and works by binding to tubulin and inhibiting microtubule assembly [26,27]. In an early phase II study performed in HER2-positive metastatic breast cancer patients who had previously received a median of 8 prior therapies, it was shown to have a median progression-free survival of 4.6 months, without any dose-limiting cardiotoxicity [28]. Hypokalemia and thrombocytopenia were the most common grade 3 or 4 toxicities, with the most common symptom being epistaxis. Other adverse events included fatigue, nausea and fever [28]. An additional phase II trial using T-DM1 in heavily pre-treated patients resulted in a median progression free survival of 6.9 months with a median duration of response of 7.2 months [29]. The EMILIA trial was the subsequent randomized phase III trial which compared standard therapy with capecitabine and lapatinib versus T-DM1 in HER2-positive breast cancer with either locally advanced or metastatic disease [30]. At a follow-up of about 19 months, patients receiving T-DM1 had an improved progression-free survival (9.6 months) compared to the standard therapy arm (6.4 months), and median overall survival was increased from 25.1 months to 30.9 months, which was statistically significant [30]. Importantly, this study was performed in women who had been previously treated with trastuzumab and a taxane. With the combination of trastuzumab, pertuzumab and docetaxel now being first-line therapy for newly metastatic patients, questions still remain: in what order should we be administering these drugs or drug combinations, and are they efficacious in a different order of administration?

These questions are becoming increasingly important as treatment for metastatic breast cancer may include multiple years of therapy. A small single institution study retrospectively analyzed HER2-positive metastatic breast cancer patients who had received T-DM1 and were subsequently administered additional anti-HER2 targeted agents after T-DM1 use [31]. The investigators report a 33% response rate to HER2-targeted agents after exposure to T-DM1, suggesting that these patients can still receive benefit from additional targeted therapy [31]. Trials are ongoing evaluating the use of T-DM1 in the first-line metastatic setting (i.e. TDM4788g, or MARIANNE, and TDM4450g), and there is a recently published phase II trial which compared T-DM1 to trastuzumab and docetaxel in the first line metastatic setting [32]. Median progression free survival increased by 5 months in the T-DM1 arm versus the trastuzumab and docetaxel arm, although overall survival was no different between the two groups [32]. Clearly further phase III studies are required to further evaluate the effectiveness of T-DM1 in the first line setting. T-DM1 has very recently received FDA-approval, making it increasingly important to evaluate the anti-tumor activity of HER2-targeted agents past progression on T-DM1.

## CNS Disease

Standard anti-HER2 therapy with trastuzumab results in a failure to adequately treat and prevent CNS progression, resulting in a growing population of HER2-positive patients with limited therapeutic options [33]. CNS metastasis is a significant issue in breast cancer regardless of HER2 status, affecting between 5–15% of patients [34,35]; this incidence is even higher in autopsy analysis to up to 30% [36], and HER2 positivity is an independent risk factor [37,38]. Patients with HER2-positive disease have even higher rates of CNS metastasis, with at least one study reporting the incidence as high as 50% of patients [39]. Additional risk factors include younger age, higher disease burden and negative hormone receptor status [40,41]. Despite the high incidence of CNS metastases, early screening for occult lesions has not been shown to improve overall survival, nor has treatment of occult disease [34,42]. Importantly, standard adjuvant trastuzumab appears to adequately prevent extra-cranial recurrence events, resulting in an increased proportion of patients with brain recurrence at first presentation of metastatic disease as identified in a meta-analysis of several multicenter, open-labels, phase III trials published in peer-reviewed journals [33].

Although trastuzumab has poor blood-brain barrier penetrance, there is evidence to suggest that alteration of this barrier with whole brain radiation allows for increased concentrations of the drug [43]. There is further pre-clinical evidence that trastuzumab acts as a radiosensitizer [44]. Multiple studies have shown that patients who have HER2-positive breast cancer, develop brain metastases, and receive trastuzumab have significantly improved overall survival than both HER2-positive patients who do not receive trastuzumab and HER2-negative patients, with a median overall survival from 12 to 24.9 months [45–48]. Other studies have shown that use of trastuzumab after diagnosis of CNS metastases lead to an improved overall survival versus patients completing trastuzumab after diagnosis of CNS metastases or receiving no trastuzumab at all [49,50].

Lapatinib shows promising activity in patients with CNS progression. In a phase 3 randomized trial of capecitabine versus capecitabine plus lapatinib in patients with locally advanced or metastatic HER2-positive breast cancer, there was a trend toward decreased CNS metastasis in the group that received lapatinib, although the difference was not statistically significant [17]. While there are no phase III trials comparing therapies for CNS metastases in HER-2 positive breast cancer patients, there are several phase I and II trials in progress testing various HER2-targeted therapy combinations with or without whole brain radiation or surgery, as outlined in a recently published review of treatment options for HER2-positive breast cancer patients who develop brain metastases [51]. A recently published single-arm phase 2 study evaluated the combination of lapatinib and capecitabine in patients with brain metastases who had not been previously treated with whole brain radiation, capecitabine or lapatinib [52]. Forty-five patients were enrolled, and 65.9% had objective CNS responses; twenty patients had had a 50–80% reduction in tumor volume, and nine had a greater than 80% reduction [52]. Another retrospective study compared HER2-positive patients with brain metastases who received trastuzumab monotherapy versus those who received both lapatinib and trastuzumab, with both groups compared against a historical control group of patients diagnosed prior to FDA approval of trastuzumab [53]. In this study, overall survival was significantly increased with the use of lapatinib in addition to trastuzumab compared to trastuzumab alone (median overall survival of 13 months and overall survival not reached, respectively) [53].

## Future Strategies

Two oral small molecule inhibitors of the HER family of receptors are in early study. Neratinib is an inhibitor of HER1, HER2 and HER4. A small phase II study evaluated neratinib in two cohorts of patients with advanced HER2-positive breast cancer, one of which had received prior trastuzumab, and the other which had not received trastuzumab [54]. At 16 weeks, the progression free survival in patients who had received trastuzumab previously was 59%, but was 78% in those who had not received trastuzumab [54]. The most frequent adverse event was diarrhea, which was worse in the patients who had received trastuzumab, and lead to dose reduction in 29% of these patients. This drug is now being tested in randomized phase II and phase III studies. Afatinib inhibits HER1 and HER2. A small phase II study of 41 patients with advanced HER2-positive breast cancer who had previously failed trastuzumab was included [55]. Of the evaluable patients, partial response was seen in 11%, and stable disease was seen in 37% [55]. While afatinib also produced diarrhea similar to neratinib, the dose-limiting toxicity was rash. This drug is also in phase III randomized testing.

Inhibition of enzymes in the phosphatidylinositol-3-kinase (PI3K) pathway, specifically the class I enzymes, is also being heavily studied, as this pathway is involved in regulation of cell proliferation, survival and apoptosis [56]. The pan-class I PI3K inhibitor, BKM120 is currently in phase I and II trials as monotherapy, as well as in combination with drugs such as lapatinib, capecitabine, trastuzumab, paclitaxel, and everolimus (NCT01132664, NCT01589861, NCT01285466, NCT01470209). BEZ235 inhibits both PI3K as well as mammalian target of rapamycin (mTOR), a serine/threonine kinase downstream of PI3K, and is also in phase I and II study as monotherapy, and in combination with trastuzumab, capecitabine and paclitaxel (NCT00620594, NCT01285466). The mTOR inhibitor everolimus is currently being studied in combination with trastuzumab and paclitaxel in the phase III setting as therapy past endocrine therapy failure (BOLERO-1, NCT00876395).

## Conclusions

While patients with HER2-positive breast cancer previously had poor outcomes, the use of trastuzumab has significantly improved their survival. As resistance mechanisms to trastuzumab have come to light, many additional targeted agents as well as drug combinations have been developed and shown to have efficacy in both disease free and overall survival. Table 1 is a proposed order of HER2-targeted therapy in advanced disease. The combination of pertuzumab, trastuzumab with chemotherapy has clearly demonstrated efficacy in the first line metastatic setting. In post-menopausal patients with HER2-positive, estrogen receptor positive breast cancer, the combination of lapatinib and letrozole can be considered as first-line therapy as well. T-DM1 has been recently FDA-approved, making it an option for second-line therapy. For further lines of therapy, it is important to note that these therapies have not been tested formally after exposure to T-DM1 or pertuzumab. CNS progression remains an important clinical challenge, and novel agents targeting intracranial recurrences are necessary to address this unmet medical need. Importantly, it has been strongly established that continuation of anti-HER2 agents is important in this patient population, despite progression on any one regimen. The additive cost of these therapies is a consideration, considering the changing healthcare field. The addition of pertuzumab to trastuzumab increases the cost of a cycle of chemotherapy by at least $4,891; the addition of lapatinib to capecitabine increases the cost of a cycle by $3942 (Medi-Span, March 2013; www.medispan.com). A cycle of T-DM1 is approximately $7211 (Genentech USA, Inc., 2013). Many additional studies are necessary and ongoing to determine the efficacy of different drug combinations, the optimal order in which to administer these agents, and the cost-effectiveness of these combinations.

## Figures and Tables

**Table 1:** Order of HER2-targeted therapy in advanced disease.

Line of Therapy^a^	Therapy for Systemic Disease	Therapy for CNS Disease
First Line	Trastuzumab combined with pertuzumab and docetaxel For patients with ER-positive disease, trastuzumab combined with letrozole is an alternative regimen	Whole brain radiation Surgical resection Stereotactic radiosurgery
Second Line^b^	T-DM1 Lapatinib combined with capecitabine	Lapatinib based therapy: combined option include capecitabine or Trastuzumab
Third Line	Trastuzumab combined with Lapatinib^c^	
Fourth Line	Trastuzumab combined with cytotoxic chemotherapy (i.e. taxane, vinorelbine)	

Abbreviations: T-DM1, Trastuzumab Ematansine; CNS, Central Nervous System

a The author support the enrollment into a well-designed clinical trial at any stage in a patient’s disease course

b T-DM1 and Lapatinib combined with capecitabine are recommended regimens in the second-line setting. However, the efficacy of these therapies after exposure to pertuzumab is known

c Trastuzumab combined with Lapatinib has not been formally tested after exposure to T-DM1 or pertuzumab.
